# Potential impact of various surface ligands on the cellular uptake and biodistribution characteristics of red, green, and blue emitting Cu nanoclusters†

**DOI:** 10.1039/d3ra03606e

**Published:** 2023-08-30

**Authors:** Kumar Babu Busi, Mathangi Palanivel, Kotha Jyothi, Fong LaiGuan Zoey, Syed Zahid, Krishna Kanta Ghosh, Bikram Keshari Agrawalla, Balázs Gulyás, Surfarazhussain S. Halkarni, Manjunatha Thondamal, Parasuraman Padmanabhan, Sabyasachi Chakrabortty

**Affiliations:** a Department of Chemistry, SRM University AP Andhra Pradesh Andhra Pradesh 522240 India sabyasachi.c@srmap.edu.in; b Lee Kong Chian School of Medicine, Nanyang Technological University Singapore 59 Nanyang Drive Singapore 636921 Singapore ppadmanabhan@ntu.edu.sg; c Department of Biological Sciences, SRM University AP Andhra Pradesh Andhra Pradesh 522240 India; d Department of Mechanical Engineering, SRM University AP Andhra Pradesh Andhra Pradesh 522240 India; e Cognitive Neuroimaging Centre, Nanyang Technological University 59 Nanyang Drive Singapore 636921 Singapore; f Protein Chemistry 1, Roche Diagnostics GmbH Nonnenwald 2 82377 Penzberg Germany; g Department of Clinical Neuroscience, Karolinska Institute 17176 Stockholm Sweden; h Department of Biotechnology, School of Technology, Gandhi Institute of Technology and Management (GITAM) Visakhapatnam Andhra Pradesh 530045 India

## Abstract

Surface functionalization has a prominent influence on tuning/manipulating the physicochemical properties of nanometer scaled materials. Ultrasmall sized nanoclusters with very few atoms have received enormous attention due to their bright fluorescence, biocompatibility, lower toxicity, good colloidal stability and strong photostability. These properties make them suitable for diagnostic applications. In this work, we intend to study the effect of surface functional ligands on their biodistribution both *in vitro* and *in vivo* organelle systems for bioimaging applications.

## Abstract 2

The effects of surface functionality on the optical characteristics of Cu nanoclusters were explored. Their *in vitro* and *in vivo* biodistribution indicated their usefulness as potential bioimaging probes.

## Graphical abstract

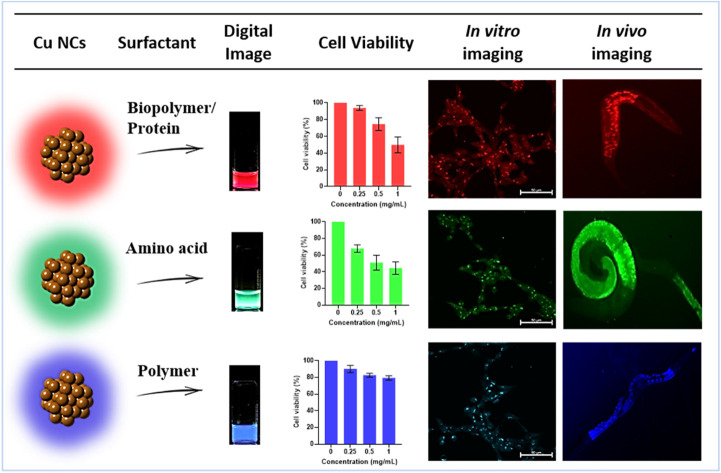

## 1. Introduction

In biomedical research, cellular uptake is one of the most vital processes for any kind of nanomaterial (NM) in various bio related applications such as drug delivery, bioimaging, diagnostics or theranostics, biosensing, *etc.*^1–4^ Most nanoparticles (NPs) are internalized into the cell *via* endocytosis.^5^ The size, shape, surface charge, composition and hydrophobicity of NPs are crucial factors determining the cellular internalization process.^6–9^ Different kinds of NMs, including organic NMs^10^ (carbon nanotubes,^2^ graphene,^11^ and carbon quantum dots^12^), noble metal (Au, Ag and Cu)^13–15^ nanoparticles (NPs) and inorganic metal or metal oxide NPs,^16,17^ have already been studied in terms of cellular uptake characteristics. Because of their wide range of applications in biomedical processes and consumer products,^18^ understanding the interactions of NPs in both eukaryotic and prokaryotic systems is critical. However, these NMs could have toxic effects on cells *via* reactive oxygen species (ROS) generation, resulting in oxidative stress.^19^ Additionally, NMs may cause cellular toxicity through mechanisms other than ROS, such as those associated with size, morphological and zeta potential alterations.^20^ There is a huge body of evidence for large metal NPs suggesting that properties such as size, shape, chemical functionality and surface charge are critical determinants for their associated biological activities.^21^ In contrast to NMs or NPs, ultrasmall metal nanoclusters (NCs) are more advantageous due to their intriguing physicochemical properties, strong luminescence, narrow size distribution, rich surface chemistry, biocompatibility, *etc.*^22,23^ Nevertheless, there are relatively few studies addressing metal NCs of sizes less than 2 nm in their biodistribution, toxicity and cellular internalization aspects, which creates high demand due to their favourable characteristics.

Fluorescent ultrasmall metal NCs (Au, Ag, Cu, Pt, Pd and Co) have garnered increasing attention as an imminent field of study because of their exceptional fluorescent properties and plethora of applications in biology and material science.^15,24,25^ These MNCs are ∼1–3 nm ultrafine sized particles that are made up of a few to tens of atoms and have a discrete electronic energy band structure that allows for quantum confinement. When the size of an electron approaches the Fermi wavelength, it exhibits molecular features such as photoluminescence (PL), highest occupied molecular orbital (HOMO) to lowest unoccupied molecular orbital (LUMO) transitions, magnetism, and molecular chirality, which make them significant and deviate significantly from its bulk counterpart.^26^ However, the luminescence property of MNCs offers optical features superior to those of conventional organic dyes, such as high intensity light absorption, large stokes shift, biocompatibility, strong photostability, and colour tunability achieved by modifying core size and surface ligands.^27^ Due to their excellent physicochemical properties, Au and Ag MNCs have been extensively investigated for a variety of applications, including bioimaging,^28^ biolabeling,^29^ biosensing,^30^ catalysis,^31^ and single molecule studies. In comparison, Cu MNCs are more attractive due to their high earth abundance and low cost. However, their tendency to oxidize easily has limited their universal applications. This has left the research community uncertain how to develop more robust biological applications, especially not knowing their biodistribution.^32^

Typically, the preparation of Cu NCs is primarily based on the bottom-up approach, whereby Cu^2+^ ions are reduced to Cu^0^ atoms *via* nucleation and the subsequent growth of clusters in the reaction solution. To stabilize these Cu NCs in the colloidal medium, various templates/ligands have been used on the surface of the metal core, such as proteins, polymers, nucleotides, DNA, small molecules, *etc.*^33–35^ All of these surface capping groups have previously been employed as good capping agents of Cu NCs that provide excellent stability and also prevent them from aggregation within the colloidal medium.^36^ In addition, surface ligands play a pivotal role in manipulating the optical properties of Cu NCs, especially the PL quantum yield (QY) and colour tunability. These optical properties have facilitated excellent opportunities in biological applications, especially in early disease diagnosis, cellular imaging, and therapeutics.^37^ However, these NCs are tiny in size and have a high surface-to-volume ratio, so it is anticipated that the surface chemistry of Cu NCs will have a substantial impact on biological outcomes as the NCs interact with biological systems. In literature, bovine serum albumin (BSA)–Au_25_ NCs showed lower renal clearance and higher toxicity compared to glutathione (GSH)–Au_25_ NCs due to their surfactant (or surface ligand) size. For Cu NCs, Busi *et al.* previously reported the synthesis of serum albumin mediated proteins adsorbed on the metal core surface of Cu NCs to study their influence on cellular uptake and biodistribution.^38^ Nair *et al.* prepared GSH–Cu NCs to investigate the influence of cell viability and perform internalization studies.^39^ Consequently, individual investigations were performed, but complete comparative studies with different sized surface ligands on cellular internalization, biodistribution and subsequent toxicity effects are limited for Cu NCs.^37^ To ensure the reliable, consistent and safe use of NCs in biomedical/clinical applications, the interactions of various surface ligands with the biological environment must be thoroughly assessed.^40,41^

The purpose of our investigation is to focus on the impact of surface capping groups on the cellular internalization and biodistribution of Cu NCs in both *in vitro* and *in vivo* models. Herein, we created different ligand attached Cu NCs that emit red, green, and blue colours (RGB) from a protein template, a single amino acid (monomer for protein) and small molecules/polymers (non-bio entity) through a simple chemical reduction method. Basic optical characterization revealed their absorption and emission properties, which confirmed the formation of RGB Cu NCs. To use these Cu NCs as diagnostic probes, we conducted cell viability studies on human embryonic kidney cell lines (HEK293T) with incubation up to 12 hours which revealed that RGB Cu NCs were biocompatible and largely non-toxic to the concentration of 0.25 mg mL^−1^. Later, we performed cellular uptake and subcellular colocalization studies with the HEK293T cell line. Interestingly, all three different colour emitting RGB Cu NCs accumulated nicely throughout the cell where the images were captured by confocal microscope in the *in vitro* model. The similar *in vivo* biodistribution imaging studies were performed on *C. elegans*.

## 2. Results and discussions

**Scheme 1:**
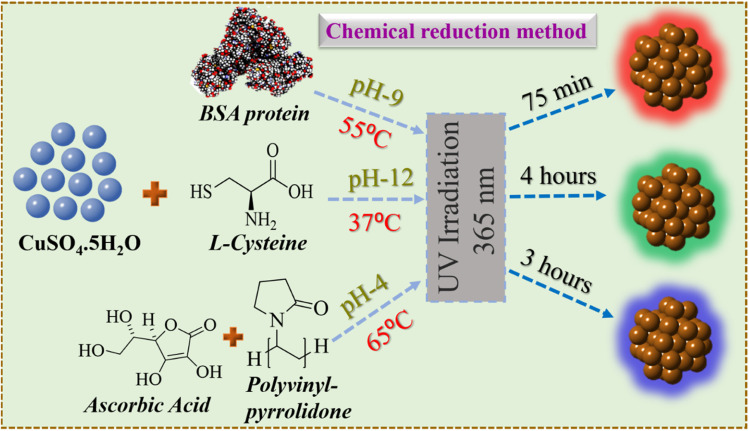
A representation of the red, green and blue colour luminescent Cu NCs prepared in aqueous medium using the same Cu precursor combined with three different surface ligands responsible for three different colour emissions.

The detailed synthesis of the red, green, and blue colour luminescent Cu NCs in aqueous medium is depicted in Scheme 1. The RGB Cu NCs were synthesised by making slight modifications to the reaction protocols described in the previous literature.^38,42,43^ In this study, we used a simple, easy, straightforward one-pot chemical reduction method to prepare RGB colour emitting Cu NCs. The general synthetic procedure of RGB Cu NCs includes CuSO_4_·5H_2_O as the precursor together with three different surface ligand combinations in individual reactions. For the red colour emitting Cu NCs, 180 mg of BSA protein was added as the surface ligand in 6 mL of water and 1 M of NaOH was used to bring the pH up to alkaline (pH 9). Then, the reaction mixture was incubated for 75 minutes at 55 °C while adding 25 μL of hydrazine hydrate (N_2_H_4_·H_2_O) as a reducing agent under continuous stirring. For the green colour emitting Cu NCs, we added 5 mg of l-cysteine as a surface ligand and reducing agent in 5 mL of water and the reaction pH was adjusted to 12. Then, the reaction mixture was incubated for 4 hours at 37 °C under vigorous stirring. For the blue colour emitting Cu NCs, we used 0.5 g of polyvinylpyrrolidone (PVP) as the surface capping group in 8 mL of water with 0.1 M of ascorbic acid (AA) as the reducing agent. The reaction mixture was incubated continuously for 3 hours at 65 °C under vigorous stirring. All three different reactions were carried out using Milli-Q water. Finally, all the reaction solutions were illuminated under UV light (365 nm) and the respective bright luminescent RGB colours were observed under the light source. Further, they were analysed using UV-Vis absorption, photoluminescence, and transmission electron microscopy (TEM). For subsequent characterization and additional experiments, the final product was stored at 4 °C. The physicochemical properties, such as size, surface morphology and respective optical features *via* different characterization techniques for the RGB Cu NCs, are clearly explained in the following sections.

**Fig. 1:**
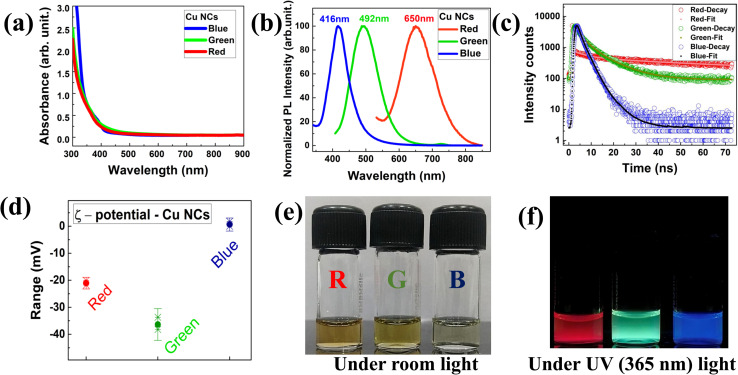
:(a) The UV-visible absorption spectra disclosed no observable sharp peaks in the Cu NCs samples, which confirmed the absence of larger sized nanoparticles. (b) The normalized emission spectra of red, green and blue colours (*λ*_max_ ∼650, 492, 412 nm). (c) Radiative decay patterns revealed the change in the recombination dynamics of Cu NCs. (d) The surface ligands present on the metal core disclosed the surface charge of the NCs. (e and f) Digital image representations of red, green and blue luminescent Cu NCs under room light and UV light (365 nm).

Fig. 1 depicts the surface functionality and optical properties of the atomically precise ultrafine sized Cu NCs. In all three-ligand mediated syntheses, the aqueous solutions of Cu NCs showed no observable sharp peak, excluding the presence of larger sized particles in the reaction medium (Fig. 1(a)). As shown in Fig. 1(b), emission maxima were observed at 416 nm, 492 nm and 650 nm for the blue, green and red colour NCs, respectively, and are attributed to the inter-band electronic transition in the emission spectrum. To examine the photoluminescence (PL) behaviour, we excited the Cu NC solutions at various wavelengths ranging from 250 nm to 400 nm. The PL intensities varied depending on their absorption cross section and a clear excitation-independent PL emission behaviour was observed without any significant emission peak shift, as shown in Fig. S1(a–c).† Interestingly, all three different RGB colour emitting Cu NCs exhibited the highest excitation maximum at ∼370 nm, which could be useful for bioimaging applications. Additionally, radiative decay measurements were performed on the as synthesised Cu NCs to gain a better understanding of the origin of the PL. As shown in Fig. 1(c), all the reaction solutions were fitted with exponential decay profiles and three different lifetime decay patterns were observed due to the surface functionalization. The PL decay values are provided in Table 1. The BSA-stabilised red emitting Cu NCs showed phosphorescence decay due to a transition from triplet to singlet state and the components were 180 ns (7.4%), 2013 ns (88.19%), and 2.44 ns (4.38%) with an average lifetime of ∼1.7 μs. The cysteine-stabilised green emitting Cu NCs time components are 2.19 ns (22.45%), 8.9 ns (70.48%), and 0.23 ns (6.97%), showing an average lifetime of ∼6.7 ns, whereas the AA, PVP-stabilised blue emitting Cu NCs time components are 1.2 ns (22.96%), 2.59 ns (66.05%), and 5.93 ns (7%), showing an average lifetime of ∼2.4 ns, with both clusters showing fluorescence decay profiles due to radiative recombination from the singlet excited state. In the above PL and radiative recombination dynamics in RGB Cu NCs, we found that the emission completely relies on ligand dependent behaviour. Taking everything into account, we confirmed that the surface ligands played a significant role in the electron transition in the energy bands, altering the optical properties of the Cu NCs. In addition, *ξ*-potential measurements were conducted to determine the surface charges for the RGB Cu NCs (∼−21 ± 0.8 mV, −36.3 ± 2.3 mV, 0.61 ± 0.9 mV) which indicate their excellent colloidal stability in the reaction solution, as shown in Fig. 1(d). The surface charges of the Cu NCs were found by taking the average of three different measurements of the *ξ*-potential values, shown in Table 2. Representative digital images of the as-prepared Cu NCs in glass vials present a strong yellow colour for the red, a pale-yellow colour for the green and a lemon-yellow colour for the blue NCs under room lighting and bright luminescent red, green, and blue colour emissions were observed under the illumination of UV light (365 nm), as shown in Fig. 1(e and f).

**Table 1:** The PL radiative decay values of RGB colour emitting Cu NCs

Sample name	Relative amplitude			PL decay (sec)			Average lifetime (sec)
	*A*_1_	*A*_2_	*A*_3_	*τ*_1_	*τ*_2_	*τ*_3_	
Red	7.43	88.19	4.38	1.80 × 10^−7^	2.01 × 10^−6^	2.44 × 10^−9^	**1.78 × 10^−^** ^**6**^
Green	22.45	70.48	6.97	2.19 × 10^−9^	8.90 × 10^−9^	2.38 × 10^−10^	**6.78 × 10^−^** ^**9**^
Blue	22.96	66.05	7	1.20 × 10^−9^	2.59 × 10^−9^	5.93 × 10^−9^	**2.45 × 10^−^** ^**9**^

**Table 2:** Zeta potential measurements of RGB colour emitting Cu NCs

Sample name	Measurement 1	Measurement 2	Measurement 3	Average (mV)
Red	−20.1	−21.3	−21.8	**−21 ± 0.8**
Green	−33.7	−37.2	−38.2	**−36.3 ± 2.3**
Blue	−0.029	0.101	1.76	**0.61 ± 0.9**

**Fig. 2:**
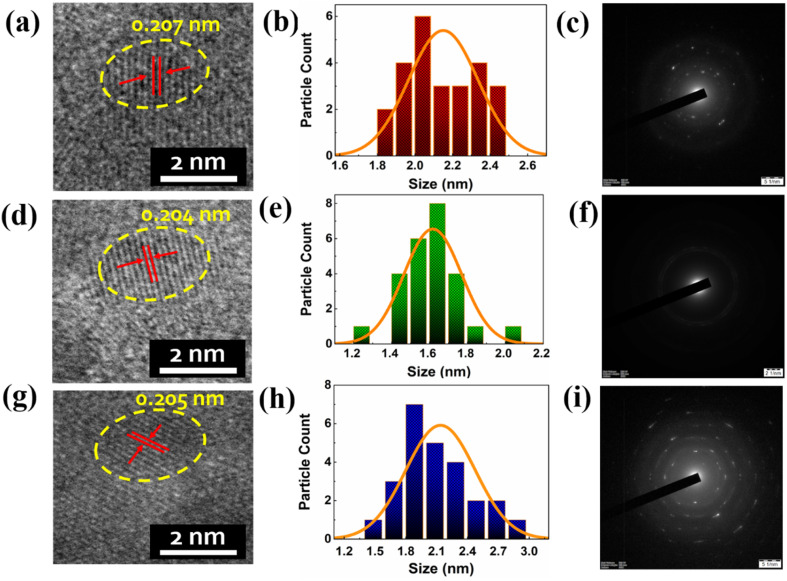
(a–c) The HR TEM image and histogram analysis showed an average size of 2.16 ± 0.25 nm, which revealed the Cu (111) plane with 0.207 nm of *d*-spacing, and the SAED patterns confirmed the low crystalline nature of red colour emitting Cu NCs due to their ultrafine size. (d–f) For green colour emitting Cu NCs, HRTEM image and histogram analysis showed an average diameter of 1.61 ± 0.21 nm, with an interplanar *d*-spacing of 0.204 nm, and the SAED pattern revealed slight amorphous behaviour. (g–i) For blue colour emitting Cu NCs, HRTEM image and histogram analysis showed an average diameter of 1.99 ± 0.55 nm, with an interplanar *d*-spacing of 0.205 nm, and the SAED pattern revealed slight amorphous behaviour.

As depicted in Fig. 2, we performed a high resolution transmission electron microscopy (HRTEM) analysis to understand the morphology, size distribution and crystalline structure of the prepared RGB Cu NCs in aqueous medium. The bright field microscopy images of Cu NCs confirmed the spherical shape arrangement of atoms associated with the protein, l-cysteine and AA, PVP surface capping agents, showing similar sizes in the three different conditions. As shown in Fig. 2(a), the average diameter of the red colour emitting Cu NCs is 2.16 ± 0.25 nm, where the interplanar spacing measurement of 0.207 nm confirmed the presence of the Cu (111) plane in the lattice structure. The histograms prepared from 25 representative tiny NCs for the RGB Cu NCS are depicted in Fig. 2(b, e and h). The selected area electron diffraction (SAED) pattern (Fig. 2(c)) suggested that the as-synthesized BSA protein mediated Cu NCs showed a finite polycrystalline nature. Fig. 2(d) presents an average diameter of the cysteine mediated green colour emitting Cu NCs of 1.61 ± 0.21 nm, with a lattice spacing measurement of 0.204 nm. As observed in Fig. 2(f), SAED analysis suggested the amorphous nature of the prepared Cu NCs due to their reduction in size. Blue colour emitting AA, PVP mediated Cu NCs showed an average diameter of 1.99 ± 0.55 nm with 0.205 nm interplanar spacing in the lattice (Fig. 2(g)). A poly crystalline structure was observed *via* SAED pattern for the Cu NCs, as shown in Fig. 2(i). All three different colour emitting tiny Cu NCs are highlighted with yellow dotted circles, as they showed spotty ring patterns without any additional diffraction spots in the SAED patterns. Furthermore, all of the Cu NCs are clearly identified, with no evidence of bulk particle formation due to aggregation.

**Fig. 3:**
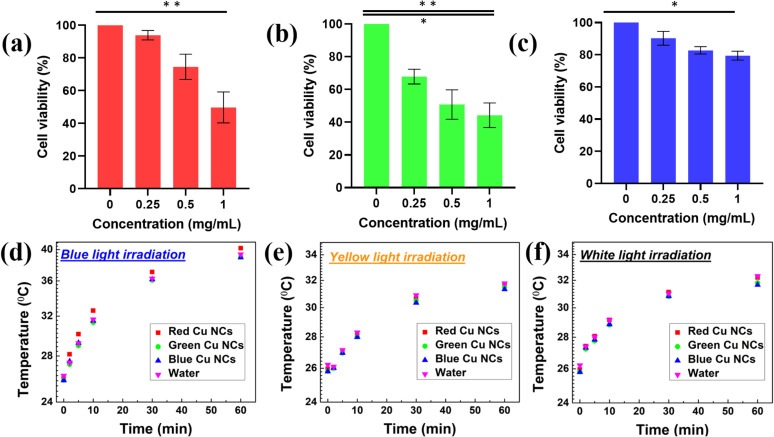
The cell viability experiments disclosed the biocompatibility of three different ligand tailored Cu NCs. (a) Protein templated red emitting Cu NCs. (b) Single amino acid l-cysteine tailored green emitting Cu NCs. (c) AA and PVP polymeric functionalized blue emitting Cu NCs. Statistical analyses of the cell viabilities with red, green and blue Cu NCs were performed with the Kruskal–Wallis test and corrected for multiple comparisons using the Dunn's statistical hypothesis testing. Data is represented as mean ± S.D. of at least triplicates. *P < 0.05, **P < 0.01 *versus* 0 mg mL^−1^ (negative control) for each colour NC. The light irradiation studies disclosed that the RGB Cu NCs could be useful for potential diagnostic applications due to their tiny size and inability to generate much heat even after one hour continuous exposure. (d) Blue colour emitting LED light source (7.08 mW cm^−2^). (e) Yellow colour emitting LED light source (0.1 mW cm^−2^). (f) White colour emitting LED light source (1.96 mW cm^−2^).

Before evaluating the influence of surface ligand size on the biodistribution, we carried out cell viability studies for the three ligand-mediated Cu NCs to check their biocompatibility using the human embryonic kidney (HEK293T) cell line, as shown in Fig. 3(a–c). The RGB Cu NCs were added to the HEK293T cancerous cell lines at different concentrations from ∼0 mg mL^−1^ to 1 mg mL^−1^ for the cell viability experiments. The as prepared Cu NCs largely showed limited toxicity towards the cell line, which maintained its normal morphology after overnight incubation. As shown in Fig. 3(a and c), the BSA protein AA and PVP ligand stabilized Cu NCs displayed a more than ∼95% survival rate for cells, while the single amino acid l-cysteine protected Cu NCs showed ∼70% viability of the cells at 0.25 mg mL^−1^ concentration. We believe that, due to the small size of the surface ligand on the green Cu NCs, the uptake into the cells is comparatively high and results in more cellular death at the same concentration of BSA protein and AA, PVP polymeric Cu NCs. As a result, the biocompatibility and extremely low toxicity of the Cu NCs showed promising potential to revolutionize diagnosis applications. To ensure this, we continuously exposed the as-prepared RGB Cu NCs to different light sources for an hour to see if they could generate heat. We used the reaction medium water (H_2_O) as the control sample and illuminated under the three different light sources of blue, yellow and white light, as shown in Fig. 3(d–f). To maintain the same concentration, the optical density was fixed (∼0.08 arbitrary units) and maintained the same for the RGB Cu NCs. The different light sources illuminated the reaction samples for approximately an hour while maintaining a constant distance (∼2 cm) throughout the measurement. Remarkably, in all three conditions, the clusters exhibited nearly identical temperature rises (∼26 °C at 0 minutes and ∼40 °C at 60 minutes) as the control H_2_O. As a result, the NCs can serve as a good luminescent *in vitro* probe because they do not generate significant heat even after one hour of continuous illumination with three different wavelength light sources. This confirms that the ultrasmall Cu NCs have minimal cellular toxicity. Furthermore, confocal microscopy was used to investigate the cellular internalization of the tailored Cu NCs in the HEK293T cell line.

**Fig. 4:**
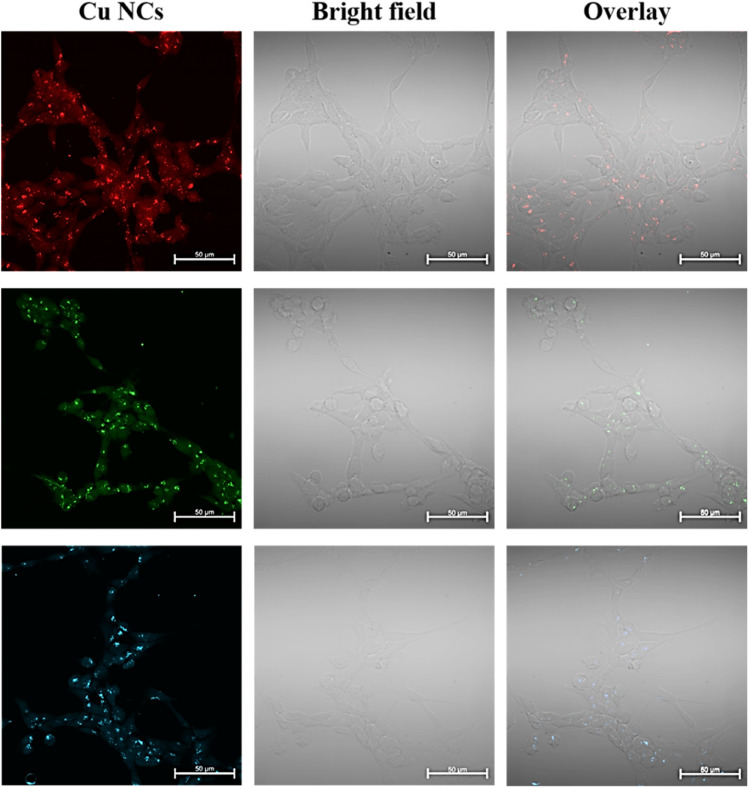
Representative confocal images of HEK293T cells treated with 0.5 mg mL^−1^ of blue, red or green Cu NCs for 4 hours. The images were captured with a Zeiss laser scanning microscope 800 (LSM800) at an oil magnification of 63×. The images depict a homogenous distribution of the Cu NCs throughout the HEK293T cells. Scale bar = 50 μm.

To understand the uptake and their intracellular morphology characteristics in more detail, we performed fixed cell fluorescence confocal microscope imaging using the concentration of 0.5 mg mL^−1^ of RGB Cu NCs after 4 hours of incubation time, as displayed in Fig. 4. In this working concentration, the cells were completely viable. Since confocal microscopy utilizes a laser and a pinhole, the resolution of the internalized Cu NCs is better. However, it was clearly observed that there was no significant alteration in the morphology of HEK293T cells after treatment with the clusters compared to the control cells. From the confocal images, it is clearly detected that the Cu NCs were efficiently taken up inside the cell: they were accumulated throughout the cytoplasm and showed bright fluorescence in the HEK293T cells. These observations indicate their biocompatible nature and extremely low toxic behaviour, suggesting the use of the Cu NCs compound as a fluorescence cell marker and delivery agent for further studies.

**Fig. 5:**
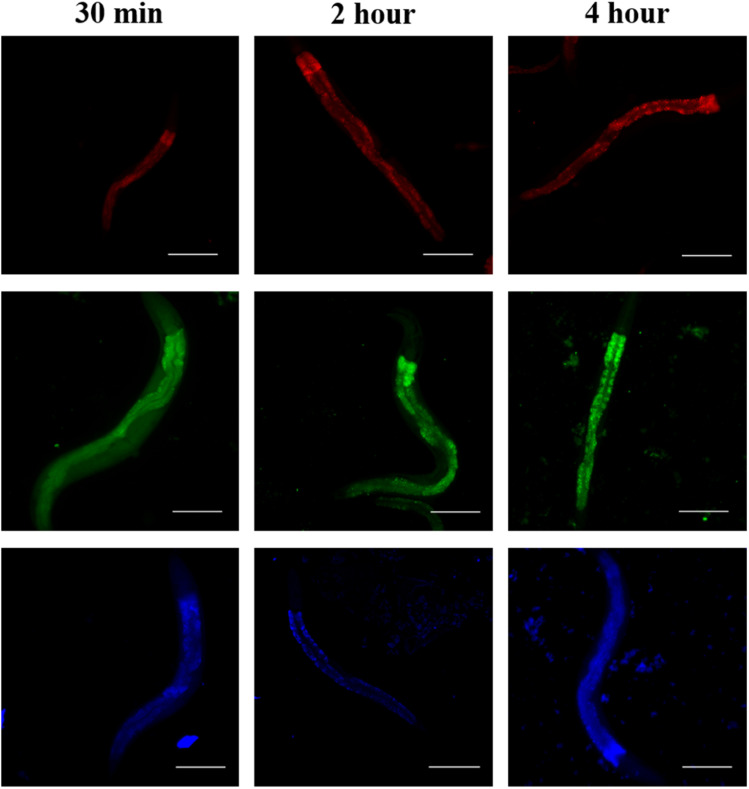
Representative images of *C. elegans* wildtype (N2 Bristol) after the uptake of red, blue, and green Cu NCs. Worms were exposed to fluorescent RGB Cu NCs for 30 minutes, two hours and four hours. The images were taken under a Nikon Eclipse Ti2 inverted microscope. Incubation time intervals from 30 minutes to 4 hours showed the even biodistribution of RGB Cu NCs and, due to the small ligand size, more fluorescence was observed with the green clusters in the worm's body. Scale bars = 100 μm.

RGB Cu NCs possess intact fluorescence properties in biological samples. To assess the cellular uptake and localization characteristics of RGB Cu NCs, we performed *in vivo* experiments to study the bio dissemination in the live model organism *C. elegans*. RGB Cu NCs were supplemented to *C. elegans* bodies in food through heat-killed bacteria to examine the survival, toxicity, and distribution. As shown in Fig. 5, the worms were exposed to Cu NCs for three different incubation time intervals from 30 minutes to 4 hours and the images were collected using an Optica inverted microscope at 10× magnification. The results showed that after 4 hours of incubation time, all the *C. elegans* were alive, which suggests that the NCs are completely non-toxic and do not affect the organism. It was noticed that the fluorescent green nanoclusters were distributed evenly and exhibited more fluorescence signal through the entire *C. elegans* body compared with the red and blue nanoclusters for all the incubation times (*i.e.*, 30 min, 2 h and 4 h). The fluorescent blue nanoclusters showed similar fluorescence behavior in the whole body. Surprisingly, the fluorescent red nanoclusters were not seen in the pharyngeal region or body of *C. elegans* within 30 min of incubation, but accumulated in the whole body after 2 h. In addition, after 4 h of incubation with the red nanoclusters, no accumulation was observed in the gonadal region, which confirms that the Cu NCs are not disturbing the reproductive system of *C. elegans*. Apparently, the size of the surface ligand played a key role in the accumulation inside the worm body. The single amino acid l-cysteine mediated Cu NCs have a tiny size in the surface ligand and the metal core which allowed them to accumulate more efficiently than the AA, PVP mediated blue emitting NCs and BSA protein-mediated red emitting Cu NCs. From all this information from both the *in vitro* and *in vivo* model organisms' accumulation, we can say that the metal core and its surface composition had an enormous influence on the cellular uptake and localization of the Cu NCs.

## 3. Conclusions

In conclusion, we successfully synthesized three distinct ligand mediated Cu NCs that emit red, green, and blue colours in an aqueous medium. Due to the change in the size of the surfactant, we observed fascinating information in the biodistribution studies. By using optical and structural characterization techniques, we demonstrated that the surface ligands had a profound impact on tuning the physical and chemical properties of the Cu NCs. The cell viability tests on HEK293T cancerous cells demonstrated the lower toxicity of the Cu NCs, even with different surface functionalities which allow them to be used in potential diagnostic applications. Using fixed cell confocal imaging, we could observe the uptake of the Cu NCs by HEK293T cells. We noticed that the percentile survivorship of the cellular organelles varied with the size of the surface ligand and metal core as well. Additionally, the *in vivo* experiments conducted in live *C. elegans* clearly demonstrate the time and ligand dependent accumulation of Cu NCs inside the whole intestinal region. Thus, exploring the impact of the surface functionalities of clusters is essential for understanding their behaviour in the complex biological environment and opens new opportunities in various biomedical or clinical applications.

## Author contributions

Conceptualization – K. B. B., M. T., K. K. G., B. K. A., P. P., and S. C.; methodology – K. B. B., M. P., K. J., F. L. G., S. Z., S. C., writing original draft – K. B. B., M. P., K. J., K. K. G., B. K. A., B. G., S. H., M. T., P. P., and S. C. review and editing – K. K. G., P. P., B. K. A., and S. C. supervision – M. T., P. P., and S. C. All authors have read and agreed to the published version of the manuscript.

## Conflicts of interest

The authors declare no competing financial interest.

## Supplementary Material

RA-013-D3RA03606E-s001
