# Supplementary material for: Potential impact of various surface ligands on the cellular uptake and biodistribution characteristics of red, green, and blue emitting Cu nanoclusters
Source: RSC Adv. 2023 Aug 30;13(37):25862–70. doi: 10.1039/d3ra03606e (PMC10466281; doi:10.1039/d3ra03606e)
Supplement: RA-013-D3RA03606E-s001 [file RA-013-D3RA03606E-s001.pdf]

Electronic Supplementary Material (ESI) for RSC Advances.
This journal is © The Royal Society of Chemistry 2023

Supporting Information

Potential impact of various surface ligand on the cellular uptake and biodistribution characteristics using Red, Green, Blue emitting Cu nanoclusters

Kumar Babu Busi,[a] *Mathangi Palanivel,*[b] *Kotha Jyothi,*[c] *Fong LaiGuan* Zoey,[b] *Syed Zahid,*[d] *Krishna Kanta Ghosh,*[b,e] *Bikram Keshari Agrawalla,*[f] *Balázs Gulyás,*[b,e,g] *Surfarazussain Halkarni,*[d] *Manjunatha Thondamal,*[c,h] *Parasuraman Padmanabhan,*[b,e*] *Sabyasachi Chakrabortty,*[a,*]

[a]Department of Chemistry, SRM University AP Andhra Pradesh, Andhra Pradesh 522240, India

[b]Lee Kong Chian School of Medicine, Nanyang Technological University Singapore, 59 Nanyang Drive, Singapore 636921

[c]Department of Biological Sciences, SRM University AP Andhra Pradesh, Andhra Pradesh 522240, India

[d]Department of Mechanical Engineering, SRM University AP Andhra Pradesh, Andhra Pradesh 522240, India

[e]Cognitive Neuroimaging Centre, 59 Nanyang Drive, Nanyang Technological University, Singapore 636921, Singapore

[f]Protein Chemistry 1, Roche Diagnostics GmbH, Nonnenwald 2, 82377 Penzberg / Germany

[g]Department of Clinical Neuroscience, Karolinska Institute, 17176 Stockholm, Sweden.

[h]Department of Biotechnology, GITAM School of Technology, Visakhapatnam, Andhra Pradesh 530045, India.

***Corresponding authors:** ppadmanabhan@ntu.edu.sg; sabyasachi.c@srmap.edu.in

Contents

A. Experimental section

i. Preparation of Cu NCs

ii. Biological studies

B. Instrumental details

C. Additional Results

1. Excitation independent emission of RGB Cu NCs

A. Experimental section

i. Preparation of different colour Cu NCs

All reactions were conducted using a three-station hot plate and oven-dried glassware, and all chemicals were used directly in the reaction without any additional purification.

Synthesis of Red emitting BSA – Cu NCs: We have synthesized the bright red colour emitting Cu NCs using BSA protein as the surface capping template by following reaction protocol as prepared by Busi et al.[1]

Synthesis of Green emitting L-Cysteine – Cu NCs: Through a slight modification to the reaction protocol as described by Soumyadip et al.,[2] we were able to create bright green luminescent Cu NCs that are mediated by a single amino acid. Initially, 19 mL of 0.1 M $CuSO_4.5H_2O$ was mixed with 5 mg of L-cysteine. After 10 minutes, NaOH (1 M) solution was introduced to the reaction to raise the pH to 12. Finally, the reaction mixture was allowed to react for 4 hours at 37°C.

Synthesis of Blue emitting AA, PVP – Cu NCs: We prepared blur colour emitting Cu NCs using AA as the reducing agent and PVP polymer as a surface stabilizing agent using a slight modification to the reaction protocol reported by Shi et al.[3] Briefly, in the glass vial, 0.5 g of PVP was dissolved in 8 mL of water, followed by the addition of $CuSO_4.5H_2O$ (0.01 M) precursor. The AA (0.1 M) reducing agent was added after 5 minutes, and the reaction solution was adjusted to pH - 4 and continuously stirred for 3 hours at 65°C.

ii. Biological studies

Cell viability assay: To evaluate the cell viability in response to the Cu NCs, HEK293T cells were seeded at a density of 15 x 10^3 cells/well in complete Dulbecco′s Modified Eagle′s Medium (DMEM) with 10% Fetal Bovine Serum (FBS) and 1% penicillin/streptomycin (PS) in 96-ell plates the day prior to treatment. On the day of Cu NCs treatment, expired media was

removed, and the cells were washed once with PBS. Then the cells were treated with red Cu NCs, blue Cu NCs and green Cu NCs at various concentrations (0 mg/mL, 0.25 mg/mL, 0.5 mg/mL, 1 mg/mL) in DMEM with 1% PS for 24 hours at 37°C in a CO_2 incubator. After incubation, a stock of Thiazolyl Blue Tetrazolium Bromide (MTT) solution (5mg/mL) was added to the wells to make up a final MTT concentration of 0.5 mg/mL. The plate was shaken at 50 RPM on an orbital shaker for 5 mins. Then the plate was incubated for 4 hours at 37°C. After incubation, the media was removed and DMSO was added to each well. The DMSO was resuspended several times to dissolve the MTT-formazan crystals. Absorbance signals from cells were measured using a Biotek Synergy H1 microplate reader. Background absorbance was subtracted from signal absorbance to normalized absorbance values. The calculated absorbance values were compared with control cells set at 100% viability.

Cellular uptake studies: To evaluate the cellular uptake of Cu NCs, HEK293T cells were seeded at a density of $1x10^5$ cells/well in complete DMEM with 10% FBS) and 1% PS in 8-wells chambered slide the day prior to treatment. On the day of Cu NC treatment, expired media was removed, and the cells were washed once with PBS. Then the cells were treated with red Cu NCs, blue Cu NCs, and green Cu NCs at 0.5 mg/mL in DMEM with 1% PS for 1 hour at 37°C in a CO_2 incubator. The cellular uptake was studied using fluorescence live cell microscopy (ZEISS Celldiscoverer 7).

Distribution studies in *Caenorhabditis elegans*: *Caenorhabditis elegans* (C. *elegans*) (wild type, Bristol strain, N2) and *Escherichia coli* (*E. coli*) OP50 were obtained from the *Caenorhabditis* Genetic Center (CGC) stock collection, University of Minnesota, St. Paul, MN, USA. Strain was maintained at 20°C on nematode growth medium (NGM) agar plates seeded with OP50 *E. coli* strain following standard protocols (Stiernagle 2006). For the distribution studies, age-synchronized worms were produced using bleaching procedure. Briefly, worms were washed off from NGM (NGM: NaCl 3 g/L; peptone 2.5 g/L; agar 23 g/L; 1M potassium

phosphate 25 mL; 1M of $CaCl_2.2H_2O$ – 1 mL; 1 M of $MgSO_4.7H_2O$ – 1 mL; 5 mg/mL cholesterol – 1 mL) plates using 5ml of M9 buffer and collected in 15ml tube. After centrifuging at 3400 rpm for 2 min, the supernatant is discarded and 5 mL of hypochlorite solution (9-12% NaOCl:1M NaOH:distilled water, 1:2:4) was added (to bleach the gravid hermaphrodites. After centrifuging the tubes for 2 minutes at 3400 rpm, the eggs at the bottom are washed thrice with M9 buffer. After final wash,eggs were transferred into a conical flask and incubated at 20 ^{o}C on an orbital shaker at 160 RPM, for 12hours at 20 ^{o}C. The L1 stage worms were dropped onto NGM plates. To prevent the growth of offspring from nematode understudy, FUDR was added to NGM plates. L4 stage worms were transferred to centrifuge tubes and washed thrice with M9 buffer. For each distribution study, 100 µL of RGB Cu NCs was added to worm pellet separately followed by 100 µL heat killed OP50 (overnight culture of *E. coli* was exposed to heat by placing the flask in water bath for 30 minutes at 55°C, and then concentrated by centrifugation) and incubate for 30 minutes, 2 hours, and 4 hours at 20 ^{o}C. After incubation period, worms were washed twice with M9 buffer and treated with 4% paraformaldehyde (4g paraformaldehyde/100 mL;distilled water; 10 mL 10X PBS/100 mL, to adjust pH to 7 1M NaOH and 10% HCl was added) to immobilize the worms. Worms were transferred onto a drop of M9 buffer placed on 3% agarose pads and coverglass was placed. Images were acquired by using, 10x-lens mounted OPTIKA B1000 fluorescent microscope.

B. Instrumental details

UV-Visible spectroscopy: To measure absorbance and PL, Greiner-96-well plates with a 200 µL capacity were employed. The microplate reader used was TECAN Spark M, and it was operated in both the absorbance and fluorescence intensity scan modes using XENON lamp source.

Transmission Electron Microscope: A high-resolution transmission electron microscope (JEOL-JEM 2100) was used to take bright-field TEM images at a high voltage of 200 kV. The copper grid (200-mesh, from TED PELLA, Inc. Redding, CA, USA) was drop-casted with 5–10 μL of BSA–Cu NCs and allowed to dry overnight under incandescent bulb (60 W) to eliminate the moisture on the copper grid. All the TEM images were analyzed with the help of ImageJ software.

Time-Resolved Photoluminescence (TRPL): Time-correlated single-photon counting (TCSPC—Horiba Jobin Yvon IBH) spectrometry was employed to obtain TRPL measurements. At repetition rates up to 100 MHz, the laser diode (Delta Diode – 425L) may produce light pulses with a standard width of 100 ps and a peak power of 230 mW. The excitation laser source peak wavelength was 420 nm, and the measurement range for sample lifetime was 6500 ns. The PL decay curves were further examined using IBH DAS6 software.

Zeta Potential: Malvern Zetasizer Nano ZS was used to measure the Cu NCs ξ-potential in electrical double-layered cells at 25°C.

Epifluorescence microscope: The epifluorescence imaging was performed with the Zeiss Cell Discoverer 7 (Jena, Germany). The light source is 385 nm LED light with emission bands 425/30 (Cu Blue); 524/50 (Cu Green); 688/145 (Cu Red)) with appropriate dichroic 405 / 493 / 610. The images were captured at 20X magnification. For *C. elegans* imaging, OPTIKA B1000 microscope with epifluorescence attachment was used and images were captured at 10X magnification.

Confocal microscope: Zeiss Laser Scanning Microscope (LSM) 800 (Jena, Germany) used for the confocal imaging at 63X oil magnification.

C. Additional results

1. Excitation independent emission of RGB Cu NCs

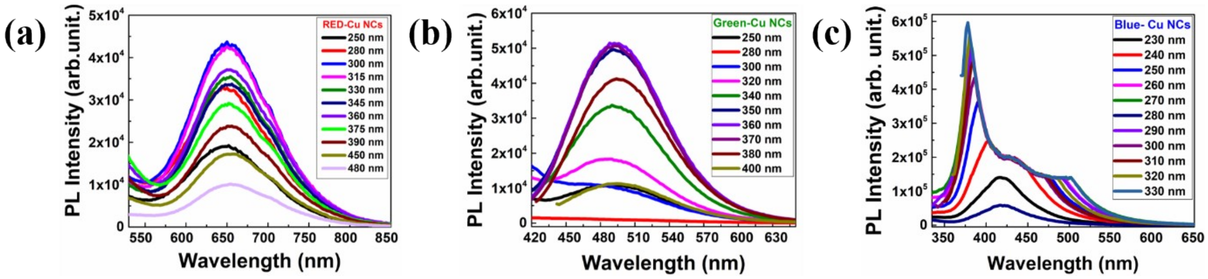

Figure S1: The PL emission patterns has been recorded for Cu NCs at different excitation wavelengths, confirmed the excitation independent emission. (a) Red; (b) Green; (c) Blue.
